# Vibration perception threshold assessments: Comparing the Staircase and von Békésy methods

**DOI:** 10.3758/s13414-025-03190-8

**Published:** 2026-01-13

**Authors:** Emanuel Silva, Nélson Costa, Isabel C. Lisboa

**Affiliations:** 1 Algoritmi Research Centre, University of Minho, Campus Azurém, 4800-058 Guimarães, Portugal; 2 Human-Technology Interaction and Robotics, CCG/ZGDV, 4800 Guimarães, Portugal; 3 NOVA School of Science and Technology, UNINOVA-CTS and LASI, NOVA University Lisbon, 1099-085 Lisbon, Portugal

**Keywords:** Vibration perception threshold, Psychophysics, Vibrotactile perception, Touch, Haptics

## Abstract

Vibration perception thresholds (VPTs) are used in fields like health, work, and traffic safety. To assess them, international standards recommend two psychophysical methodologies: the von Békésy method, or variants of the up and down algorithm, for example, the Staircase method. However, their impact on the results of threshold measurement has not been explored much, including on assessments at frequencies > 250 Hz. Thus, this study compared VPT results obtained by the two methods to determine what differences could be found between them. Using the Staircase (3 Down/1 Up rule) and von Békésy methods, VPT assessments were conducted at the pulp of the right index and ring fingers of 30 healthy subjects, at vibration frequencies of 250 Hz, 375 Hz, and 500 Hz. Analysis revealed significant differences between the mean VPTs across the three frequencies, regardless of method (VPTs increasing as frequency increased). Significant differences were also found between results from both methods (lower thresholds with Staircase). Significant differences were further found between VPT results assessed at the index and ring fingers (lower on the latter). However, this was only verified in results obtained using the Staircase. These findings highlight the importance of method selection during experimental design and when interpreting or comparing findings between studies using different VPT assessment techniques. All data related to the study presented in this paper is available at the Open Science Framework (OSF), at the following URL: https://osf.io/3uqsb/overview?view_only=03e228c15a274cb781045e3b61ccb052. This study was not preregistered.

**Supplementary information:**

The online version contains supplementary material available at 10.3758/s13414-025-03190-8.

## Introduction

Haptics – our sense of touch – is integral to our everyday experiences. This sense enables us to feel the squeeze of a hug, the vibration coming from a phone’s notification, and the contours of the button to change the radio station while we drive, all without having to rely on our eyes and ears. After all, the skin is one of the largest human organs, and touch is the earliest of our five senses to develop in the womb (Hooker, 1952, as cited in Montagu, 1984).

Despite this, most contemporary interfaces, like dashboards and smartphones, tend to favor other interaction modalities, such as visual and audio, over touch (MacLean, 2008). Users, however, seem to appreciate when their Human-Machine Interfaces (HMIs) can generate haptic feedback, as haptic interaction is a naturally simple way to receive and process information. Furthermore, this interaction modality is available to blind and deaf individuals as well, making it more inclusive overall (Basdogan et al., 2020). Thus, well-designed tactile interfaces can improve our interactions with technologies and drive product sales (Jansson-Boyd, 2011). As such, research and technology companies are gradually focusing on improving haptic feedback in HMIs once again. Commonly, such interfaces employ vibrotactile stimuli, as there is a wide number, size, and variety of actuators that can create simple vibrations, making it easy to incorporate them into various devices (Culbertson et al., 2018).

Devices that employ vibrotactile feedback have been developed across various contexts. In medicine, for example, Bark et al. (2013) introduced a haptic feedback system to a surgical machine system, which captured the vibrations sensed by the tools on the patient’s side and reproduced them on the controls that the surgeon used during the operation. In the context of interpersonal communication, in turn, Plaisier et al. (2020) employed a custom-made device to assess if participants could be taught to recognize letters and words actuated as vibrations following the vibrotactile Morse code alphabet. In a traffic safety context, Lisboa et al. (2024) introduced a haptic motorcycle jacket and a pair of haptic GPS (Global Positioning System) gloves to transmit tactile alerts and warnings to riders while driving under various vibration conditions. Yang and Roofigari-Esfahan (2023), in turn, assessed the usability of using vibrotactile feedback, integrated into the equipment of road construction workers, to deliver warnings regarding incoming traffic. Finally, haptic cues, such as vibrations, have also been proposed as an efficient method to inform car passengers of upcoming road conditions (e.g., warnings of turns, bumps, or stops), helping to reduce motion sickness in autonomous cars (Pereira et al., 2024).

However, to properly implement this feedback and allow it to provide richer sensations, haptic experience designers (i.e., hapticians) need to ensure that users can perceive these vibrations and that such vibrations are not uncomfortable. For that, information coming from psychophysics studies measuring vibration perception thresholds is essential (Schneider et al., 2017).

The vibration perception threshold (VPT) is one of the most relevant psychophysical metrics related to perceiving vibrations. This threshold refers to the minimum amount of amplitude required to perceive a vibration (Griffin, 1990; Morioka & Griffin, 2002). Determining VPTs can offer valuable insights into the ranges of frequencies and amplitudes that are perceptible without causing discomfort for different users. As VPTs are influenced by various factors, such as the vibration’s frequency, the contact location between the vibrating object and skin, and a person’s age, among others (Gandhi et al., 2011; Griffin, 2012; Silva, Lisboa, & Costa, 2024a), having access to more information regarding how different combinations of these can affect thresholds can help hapticians better customize the vibration intensity of their devices to fit the different contexts. For example, older individuals tend to be less sensitive to vibrations and thus require them to be more intense to perceive them, i.e., older individuals have higher VPTs than younger ones (Dahlin et al., 2015; Ekman et al., 2021). Since their perception thresholds are higher, the amplitude necessary for vibrations to cause them discomfort is higher as well, as discomfort is only expected to occur at magnitudes above perception level (Morioka & Griffin, 2006, 2009). However, if vibration intensities in a device aimed at multi-generational users are set up only taking older users and their sensitivities into account, said intensities may end up being perceived as uncomfortable or annoying by younger users with lower VPTs and thus lower discomfort thresholds (Morioka & Griffin, 2006, 2009), which may lead to them abandoning the device. VPT measurements also play a crucial role in the early detection of several pathologies, such as diabetes (Ising et al., 2018; Lindholm et al., 2019) and hand-arm vibration syndrome (Gerhardsson et al., 2013; Taylor, 1993). Thus, standards for measuring VPTs, such as ISO 13091-1 (International Organization for Standardization, 2001), and respective normative data, like ISO 13091-2 (International Organization for Standardization, 2003), were created. These standards and respective normative data sets have important clinical applications, with various studies testing and contributing to their expansion (e.g., Ahn et al., 2013; Ekman et al., 2021; Lindsell & Griffin, 2003; Lundström et al., 2018).

According to the International Standards for assessing VPTs, ISO 13091-1 (International Organization for Standardization, 2001), psychophysical methodologies utilized to this end should make use of either constant or intermittent stimuli, such as the case of the von Békésy method, which employs constant stimuli, or a variant of the up and down algorithm, such as the Staircase method, which employs intermittent stimuli. In the von Békésy method, participants experience a constant vibration with an amplitude that changes based on their moment-to-moment responses. In the Staircase method, in turn, participants experience short vibration trials, with amplitude adjustments occurring between trials based on whether they perceived the previous vibration. Of the two approaches, ISO 13091-1 points to methodologies following the up and down algorithm, i.e., those employing intermittent stimuli, like the Staircase, as preferable for conducting VPT assessments, as the stimuli interval between trials not only allows participants to contrast the applied stimulation against any existing background vibration, but also decreases the likelihood of a temporary threshold shift occurring due to the previous presence of a stronger stimulus, as these two situations can cause errors in VPT measurements (International Organization for Standardization, 2001). However, in publications involving the study of VPTs, the von Békésy method is the one most often used (Gandhi et al., 2011; Silva, Lisboa, & Costa, 2024b). This selection might be due to this method’s time efficiency when compared to methodologies like the Staircase, as it not only allows for either a hard or a soft time limit to be imposed for each condition, but also removes the wait times implicit in methods with intermittent stimulation. This difference allows researchers to gather data from more conditions or repetitions over a single session, for example, but also keeps the time investment required from both researchers and participants low (Gandhi et al., 2011; Silva, Lisboa, & Costa, 2024b).

One potential issue that might arise from using the von Békésy method, however, is that it only estimates thresholds for 50% probability–chance-level probability. This means that if ten same-frequency vibrations are generated with an amplitude set to a threshold level gathered with this method, it can be expected that about five of those vibrations will be perceived by an observer. The Staircase method, in its basic form following a 1 Down/1 Up rule (i.e., the amplitude of the stimulus is decreased after one “Not detected” answer, and increased after one “Detected” answer), in turn, also estimates thresholds for 50% probability. However, these probabilities can be changed by changing the rules of increases and decreases. For example, by using a 3 Down/1 Up (3D/1U) rule, this probability becomes 79.4% (Morioka & Griffin, 2002), increasing our certainty that vibrations with amplitudes set to thresholds assessed with this method will be perceived.

Although there were differences in the estimated threshold probabilities between the Staircase method, following a 3D/1U rule, and the von Békésy method, Morioka and Griffin (2002) observed that when comparing VPT results at frequencies of 16 Hz, 31.5 Hz, 63 Hz, and 125 Hz, measured at a fingertip, the thresholds obtained using the Staircase method were generally lower than those from the von Békésy method. However, these differences were statistically significant only at 125 Hz. This frequency was the highest the authors studied, and, to the best of our knowledge, other studies have not yet investigated if significant differences might also be found between VPT results obtained by these two methods at frequencies above 125 Hz. Furthermore, the authors also employed different magnitudes of change between both methods, with the von Békésy method following a constant change rate of 3 dB/s, while the Staircase method followed a constant step size of 2 dB. Similar results were also found by Maeda (1992; as cited in Morioka & Griffin, 2002), who compared VPTs assessed at 125 Hz with the von Békésy method and variations of the Staircase method, both targeting 50% probability, and also found thresholds to be higher when obtained through the von Békésy method.

As the amplitude of vibrations produced by various types of vibrotactile actuators is related to the frequency of the produced vibration itself (with higher frequencies allowing for higher amplitudes to be produced), it is important to provide hapticians not only with VPT data assessed from various higher frequency vibrations, but also with information to guide them when navigating through VPT results from various studies, methods, and body locations. This guidance is essential for interpreting how these factors interact with a user’s perception and comfort across different devices and applications. Additionally, differences in methodologies, such as the type of psychophysical procedure used or the specific anatomical sites tested, can lead to variability in threshold data. It is, therefore, essential to investigate these differences and understand the advantages and disadvantages of the two methods when they are used to assess VPTs.

In the present study, we compared VPT results assessed using the Staircase and von Békésy psychophysical methods, at three frequencies above 125 Hz, namely 250 Hz, 375 Hz, and 500 Hz. Furthermore, we collected VPTs from two locations: the finger pads of the right index and ring fingers. The right index finger was selected due to being the most prevalently studied hand location in various prior VPT assessment studies, while the right ring finger, in turn, was chosen due to being one of the least studied (Silva, Lisboa, & Costa, 2024b).

## Method

### Participants

Thirty-two (*N* = 32 (*n* = 30)) healthy participants were recruited from around the University of Minho’s Guimarães (Portugal) campus, through direct contact or recruitment email. Two participants were excluded from analysis due to technical problems. One exclusion was due to an initial lack of hand-temperature control methods (cf., *Data gathering process* section), while the other was due to a software error resulting in data not being recorded during one of the study’s experimental phases. Data from the remaining 30 participants (18 male) were included for analysis. Prior studies conducting VPT assessments with either of these two methodologies have employed similar sample sizes (for a review, see Silva, Lisboa, & Costa, 2024b). Participants were aged between 22 and 46 years (mean 31.67 ± 5.49 years), with an average height of 1.73 ± 0.07 m (1.58–1.98 m), and an average weight of 73.70 ± 10.72 kg (50–120 kg). Participants’ handedness was assessed through the Portuguese version of the Edinburgh Handedness Inventory (Espírito-Santo et al., 2017). An overview of the participants’ characterization is presented in Table 1. Data were collected between December 2023 and April 2024.

**Table 1:** Participant characterization

		Number of participants (*n* = 30)
**Handedness**		
	Right-handed	28
	Ambidextrous	1
	Left-handed	1
**Suffers from pathologies affecting skin sensibility**		0
**Smoking habits**		
	Non-smoker	27
	Smoker	3
**Daily exposure to high-frequency vibrations**		1
**Previous trauma to hand(s), arm(s), or head**		7
**Scar(s) on finger(s) or palm(s)**		1
**Taking medication**		10

### Apparatus

**Fig. 1:**
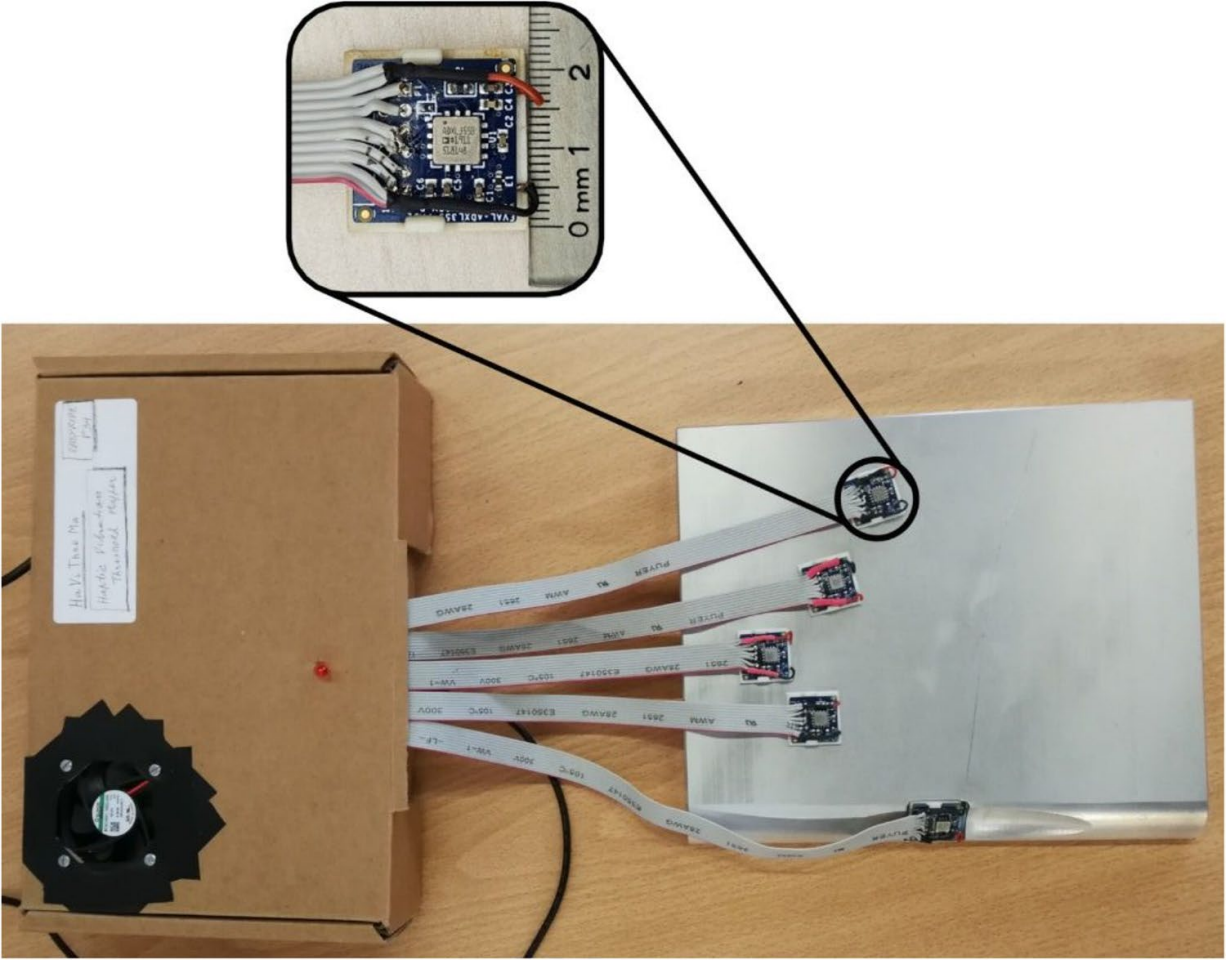
HaViThreMa testing platform. Top: Top view of a Piezo Cradle with side measurement. Bottom: Base platform with five piezo cradles in position for the five fingers of the right hand

Assessments were carried out using the Hand Vibration Threshold Mapper (HaViThreMa) testing platform (Silva, Lisboa, Matias, et al., 2024). This tool can generate vibrations on up to seven different hand locations, either simultaneously or individually. These vibrations are generated by piezoelectric actuators (piezo, upgraded to TDK 1204H018V060 models since Silva, Lisboa, Matias, et al., 2024) following pre-programmed procedures. Due to its design, vibration amplitudes on the HaViThreMa are controlled as a percentage of the power input delivered to each actuator rather than specific amplitude values being input and manipulated. Each piezo is placed at the bottom portion of a structure called a “Piezo Cradle” (Fig. 1), with one cradle per actuation location. Each cradle also houses a MEMS accelerometer (ADXL355) placed above the actuator, which measures the acceleration of the vibrations generated by the piezo. The accuracy and precision of the ADXL355 accelerometers were validated by comparing their measurements to those from a calibrated vibration analyzer (Quest VI-400 Pro) coupled with a reference analog accelerometer (Dytran 3023). Sensors were mounted on a vibration exciter (Brüel & Kjær Type 4809) operating at a constant amplitude across a frequency range of 50–650 Hz. From this, the ADXL355 was shown to exhibit a measurement profile closely matching the reference system, with a near-identical polynomial fit across the frequency spectrum, showing lower measurement variance, which indicates the high reliability of these accelerometers. This comparative procedure served as a validation of the accuracy of the ADXL355 accuracy and thus of the HaViThreMa platform’s measurement integrity (for more information on this tool, please consult Silva, Lisboa, Matias, et al., 2024).

During assessment procedures, the participant’s skin rests on top of each accelerator’s PCB and MEMS components, causing a skin indentation of ≈ 0.2mm. Each piezo cradle, in turn, rests atop a 2 cm thick metallic heavy plate (base platform), which not only prevents vibrations from spreading to other skin locations, but also reflects the downwards vibration energy back up towards the skin, using the cradle’s structure as a medium.

HaViThreMa has its own custom-made Graphical User Interface (GUI), which keeps users updated regarding the current trial’s vibration frequency (Hz) and amplitude (% of power inputted, and outputted g rms), the actuated location, the current number of reversals achieved (i.e., changes from amplitude increase to decrease, and vice-versa), the number of conditions that have been completed and how many are there in total, and the current trial number. While the accelerometers employed in the HaViThreMa report acceleration (*g rms*) as their default (as displayed in the GUI), all VPT data outputted to the answer files was converted to m/s^2^ rms and dB relative to 10^−6^ m/s^2^ rms (henceforth referred to simply as dB), using equations $1$ and $2$:

1 $$amp=g*OneG$$

2 $$dB=20*{\mathrm{log}}_{10}\left(\frac{amp}{refdB}\right)$$

where *amp* describes vibration amplitude, in m/s^2^; *g* describes acceleration (*g*); *OneG* describes the gravity constant of 1 g = 9.90665 m/s^2^; and *refdB* describes 10^−6^ m/s^2^, as stipulated in ISO 13091-1 (International Organization for Standardization, 2001).

### Procedure

VPT data were collected for three frequencies (250 Hz, 375 Hz, and 500 Hz) at two locations of the right hand (pulp of index finger, Index; and pulp of the ring finger, Ring), resulting in six total experimental conditions (Index–250; Index–375; Index–500; Ring–250; Ring–375; Ring–500). All six conditions were implemented in both the von Békésy and Staircase methods. The order of conditions was randomized for each method, while the order of methods was counterbalanced between participants, with half of the participants starting with the von Békésy method and the other half with the Staircase method, to avoid any learning or order effects. In both methods, all conditions were presented once. Piezo cradles were placed under the subject’s five fingers, and they were not informed about which locations assessments would be conducted at. Additionally, data were also collected at each trial regarding how much time had passed since the start of the condition. These data were used to calculate how much time participants took to complete each trial (time in seconds to complete, or TTC).

Before the experiment, participants underwent 1 (Staircase) or 2 (von Békésy) training conditions (data not recorded). A vibration frequency of 450 Hz was selected for the training conditions of both methods, as it was not among the three frequencies used in the experimental phase, is in the tested frequency range of 250–500 Hz, and was high enough to be perceivable by all participants at 100% amplitude. For the training phase of the von Békésy method, vibrations were delivered to the right thumb and middle finger (randomized order). For the training phase of the Staircase method, in turn, vibrations were delivered to the right middle finger only, for brevity.

The elaboration of the procedures for the von Békésy and Staircase methods were based on those presented in Morioka and Griffin (2002), with adjustments implemented to better fit the HaViThreMa’s capabilities. To improve results from both methods, an initial change rate/step size of 5%, and a follow-up change rate/step size of 2%, were used for both methods. Due to hardware and software limitations, a change rate of 1.25 s between trials was the smallest rate that could be implemented for the von Békésy method, as shorter rates proved unreliable during pilot testing. For both methods, a reversal was considered to have occurred whenever the participant’s “not detected” and “detected” answers triggered a change from stimulus amplitude going from increasing to decreasing, or vice-versa*.*

### Von Békésy method

In the von Békésy procedure, a vibration was constantly actuated while a condition was running. During a condition, participants were asked to tap the Down-arrow key as soon as they felt (“Detected” answer) and stopped feeling (“Not detected” answer) a vibration. Participants were informed that if they felt unsure regarding what their last answer had been, at any point during a condition, they were allowed to press the answer button again.

**Fig. 2:**
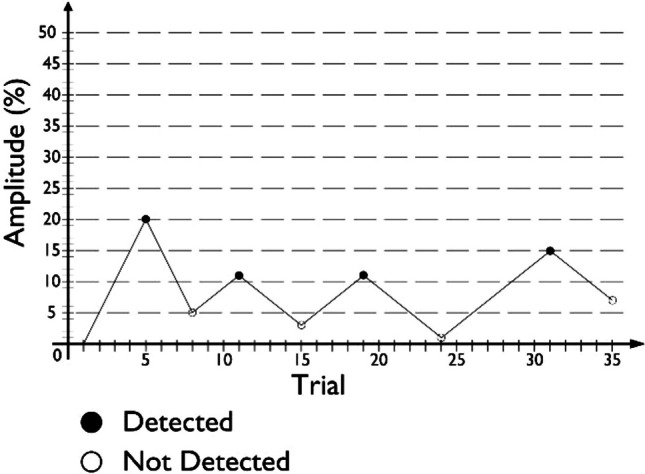
Example of a condition’s procedure following the von Békésy method used in this study

The stimuli amplitude of each condition started at 0% (i.e., no vibration), and increased by 5% every 1.25 s until participants answered “Detected,” marking the first reversal. Following this, stimuli amplitude began to decrease by 5% every 1.25 s. After a “Not detected” answer, stimuli amplitude began to increase once again (i.e., after the second reversal occurred), now with a reduced change rate of 2% every 1.25 s. For the remainder of the condition, this change rate was in effect, with amplitude decreases occurring after every “Detected” answer, and increases occurring after every “Not detected” answer. An example of a condition’s procedure, following this method, is given in Fig. 2.

While a condition was running, a red LED was turned on. When switching from one condition to the next, this LED would blink to inform participants of this occurrence, so that they would not press an answer button during this occurrence. To shorten the training phase of this portion of the study, only the initial change rate of 5% every 1.25 s was used, with all other procedure aspects remaining the same as during the experimental phase.

Conditions with this method were set to end after one of three events occurred:

- 30 s or more passed since stimuli onset, and at least eight reversals occurred (172 occurrences);
- stimuli amplitude remained at 100% for 15 s, with the last answer being “Not detected” (0 occurrences); or
- stimuli amplitude remained at 0% for 15 s, with the last answer being “Detected” (eight occurrences).

Thresholds were calculated as the average of all reversal pairs, excluding the first two, for all conditions where more than eight reversals occurred. Data from incomplete pairs were excluded from these calculations to not bias threshold results, for example, if a condition’s last reversal was the 13th, said reversal was excluded and calculations were done with data from reversals 3 to 12. TTC each condition, in turn, was calculated as the time interval between the answer to the first and last trials, for each condition where more than eight reversals occurred.

### Staircase method

**Fig. 3:**
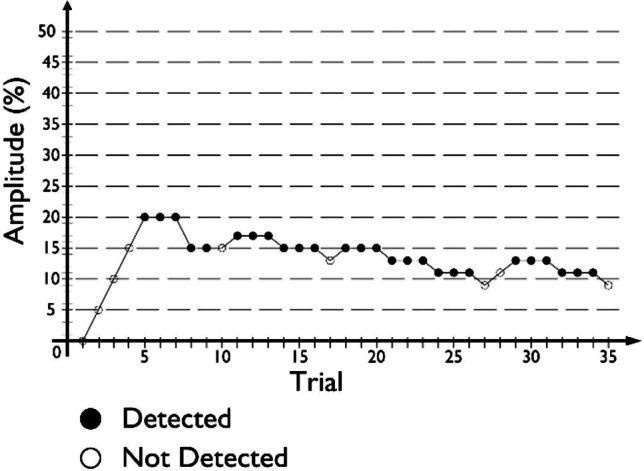
Example of a condition’s procedure following the Staircase method (3 Down/1 Up rule) used in this study

In the staircase procedure, a vibration stimulus was actuated for 2 s during each trial of a condition. A red LED turned on 1 s after the stimulus ended, indicating to participants to input their answers, using the Up-arrow key for “Detected” answers and the Down-arrow key for “Not detected” answers. The following trial started 1 s after each answer. The stimuli amplitude was increased after one “Not detected” answer and decreased after three consecutive “Detected” answers (3 Down/1 Up rule). For each condition, stimulus amplitude started at 0% and was changed in step sizes of 5% until the second reversal occurred, after which the step size was reduced to 2%. To shorten the training phase of this portion of the study, a 1 Up/1 Down rule was implemented, with only the initial step size of 5% being in effect throughout. All remaining procedural aspects were the same as during the experimental phase. An example of a condition’s procedure, following this method, is given in Fig. 3.

Conditions with this method ended after one of three events occurred:

- eight total reversals occurred (178 occurrences);
- three “Not detected” answers were given with stimulus amplitude at 100% (0 occurrences); or
- three “Detected” answers were given with stimulus amplitude at 0% (1 occurrence).

Additionally, in one occurrence, condition Ring-250 for one participant was left unfinished due to a software issue that occurred during the session. Thresholds were calculated as the average of all reversals, excluding the first two, for all conditions where eight reversals occurred. TTC each condition, in turn, was calculated as the time interval between the answer to the first and last trials for each condition where eight reversals occurred.

### Data-gathering process

Participants took part in one session lasting around 50 min, conducted in a quiet room. After being given a short introduction to the study and its context, participants signed the informed consent form, which was followed by a questionnaire with the following information: sociodemographic characteristics; the Portuguese version of the Edinburgh Handedness Inventory (Espírito-Santo et al., 2017); the presence of pathologies that might affect their perception of vibrations on the hands; if they had suffered any previous trauma to their hand(s), arm(s), or head; daily exposure to high-frequency vibrations; and if undergoing any medication. Afterward, skin temperature at the pulp of their right index and ring fingers was measured using a Brüel and Kjær Indoor Climate Analyzer Type 1213, with the Surface Temperature Transducer (MM 0035) equipped. If the skin temperature of either finger was below 20 ºC, participants were instructed to warm their hands using a small heater, until the skin temperature of both fingers was equal to or above 20 ºC, as lower finger skin temperatures are known to impact VPT assessments negatively (Ekman et al., 2021; Harazin et al., 2013).

Following this, participants sat at a table (where the HaViThreMa was placed) and were equipped with noise-canceling headphones (Sony WH-1000XM4). Participants were asked to leave their left ear physically uncovered during the training phases to hear the instructions correctly. Both ears were physically covered by the headset during the experimental phases. These headphones were connected to the researcher’s laptop and had noise-canceling set to maximum, with all other options, such as voice activation, disabled. During the two experimental phases of this study, white noise was played through these headphones to mask the sound produced by the HaViThreMa’s actuators, ensuring that auditory cues did not influence participants’ responses.

**Fig. 4:**
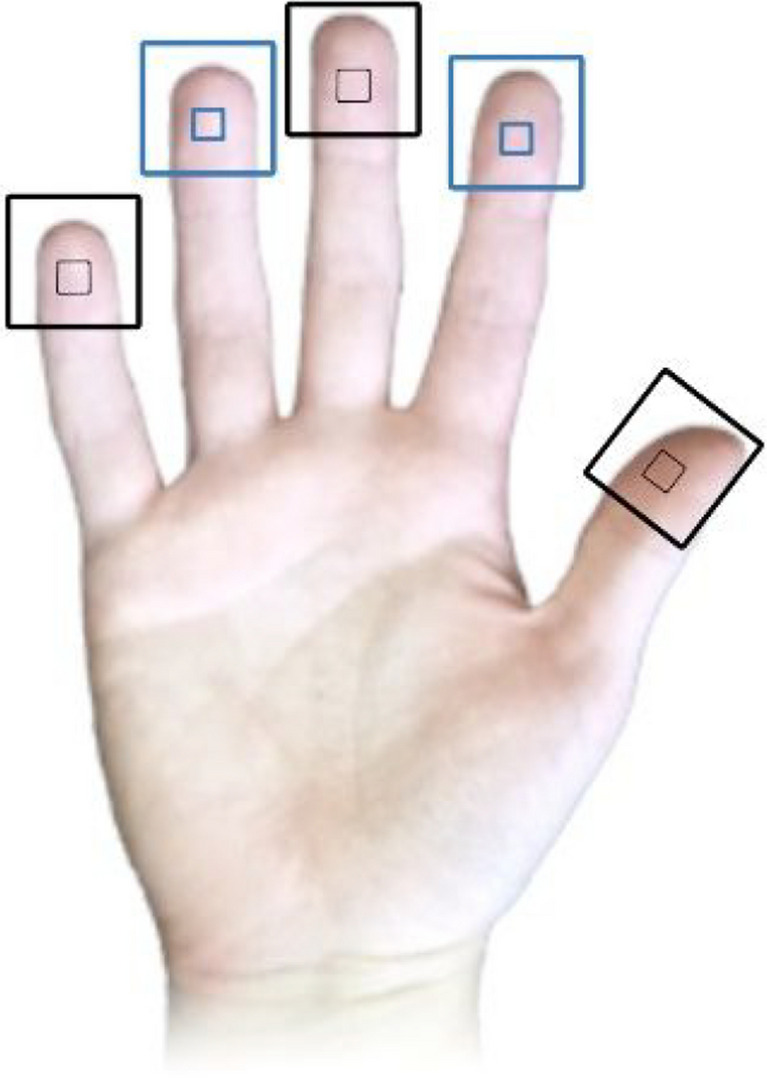
Placement of piezo cradles on the hand. Black markers indicate locations where vibration perception thresholds (VPTs) were not assessed, while blue markers indicate locations where VPTs were assessed

With help from the experimenter, participants placed their right hand on the HaViThreMa (see Fig. 4 for locations). Participants were instructed to ensure that the MEMS component of each accelerator was placed under the fingerprint’s swirl and to let their fingers rest on top of each cradle in a natural resting position, exerting neither more nor less pressure than when normally resting. They were also free to spread out or bring their fingers near each other, for comfort, but were specifically instructed not to lift them from the Piezo Cradles while conditions were running. The contact pressure between the participant’s skin and each piezo cradle was not measured throughout this study, as the HaViThreMa does not possess this feature.

**Fig. 5:**
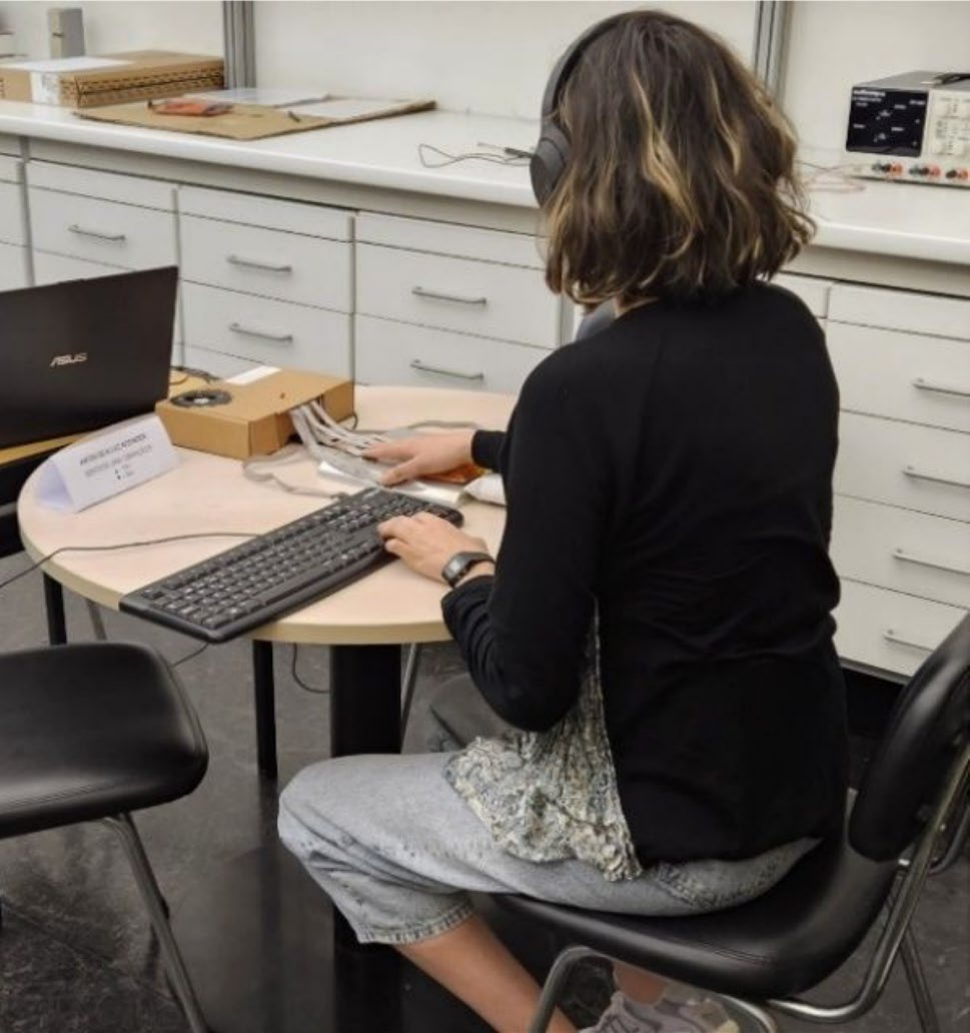
A participant during the study with HaViThreMa

A cotton-filled bag supported participants’ arms and a reusable hand warmer (Lifesystems©, product code 42450) was activated and placed under their palm to help maintain their hand’s skin temperature throughout the session. Before starting the procedure, participants were free to adjust the chair’s height to a level comfortable for them. A keyboard was placed on the table, near their left hand, which they were instructed to use to input their answers throughout the procedure. To aid participants in remembering which keys to press for which answer during each methodology, a paper memo (in Portuguese) was placed in front of them, with information regarding the Staircase method on one side and the von Békésy method on the reverse (see Fig. 5)

After being comfortably installed, participants were informed they could ask for a break at any point during the study. They were then given instructions regarding how to provide answers for the assessment methodology that would be used during the first half of their session and went through that respective method’s training phase. The training phase of either method could be repeated up to three times, and clarifications regarding the task were given when asked, before moving on to the experimental phase. During either training phase, the headphone’s noise-canceling was active, but no white sound was played, to ease communication and familiarize participants with the noise-canceling feature.

Before the start of an experimental phase, participants covered their left ear with the headphones, through which a white noise sound was then played, and were asked to adjust the volume of this sound, after which the experimental phase began. After concluding all conditions for one method, participants took a break before the start of the next phase. This phase began with the researcher explaining the instructions for the procedure following its respective assessment methodology. If participants removed their hands from the HaViThreMa during this break, the experimenter would help them place the Piezo Cradles in their respective locations once again.

After the end of the second experimental phase, finger skin temperature measurements were taken once again from the pulp of the right index and ring fingers. Following this, the session was concluded. Table 2 presents the mean, standard deviation (SD), and range for the finger skin temperatures of the right index and ring fingers, at the start and end of the session, as well as the difference between these temperatures.

**Table 2:** Right index and ring finger skin temperature measurement results

	Start temperature ºC (SD)	End temperature ºC (SD)	End–Start temperature ºC
Index	29.35 ± 5.33 (20.8–38.8)	29.08 ± 4.53 (18.1–34.6)	−0.27
Ring	29.49 ± 5.37 (21–35.3)	29.16 ± 4.57 (18.6–34.8)	−0.33
Grand average	29.42 ± 5.35 (20.8–38.8)	29.12 ± 4.55 (18.1–34.8)	−0.30

### Transparency and openness

All data, analysis code, and research materials are available on the Open Science Framework repository at the following link: https://osf.io/3uqsb/overview?view_only=03e228c15a274cb781045e3b61ccb052. All analyses were conducted using R (R Core Team, 2023) through RStudio (Posit team, 2024), with the “nlme” (Pinheiro et al., 2023; Pinheiro & Bates, 2000), “ggplot2” (Wickham, 2016), “dplyr” (Wickham et al., 2023), and “car” (Fox & Weisberg, 2019) packages. This study was not preregistered.

VPT assessments and TTC data were individually organized according to the psychophysical method (Staircase method or von Békésy method) and experimental conditions. Linear mixed-model analyses were performed to investigate within-person differences over all three frequencies, the two methods, the two finger locations, and both genders. This model was selected as it does not require complete data sets, allows data from non-independent samples, and allows for the modeling of variability in the form of regression slopes (Field et al., 2012). Maximum-likelihood estimation (ML) was used on all fitted models, except for the *Final_VPT_Model* and *Final_TTC_Model* (see *Results* section for model description), which were fitted at the end of the process using restricted maximum-likelihood estimation (REML) instead to provide unbiased estimates of the variance parameters. The optim optimizer was used on all linear mixed models. Missing data (i.e., conditions where participants did not achieve a minimum of eight total reversals) was set to be excluded, identified in data as “NA” (Field et al., 2012). Finally, 95% confidence intervals (CI) and p-values were calculated using a Wald t-distribution approximation.

## Results

### Vibration perception thresholds (VPTs) overview

**Fig. 6:**
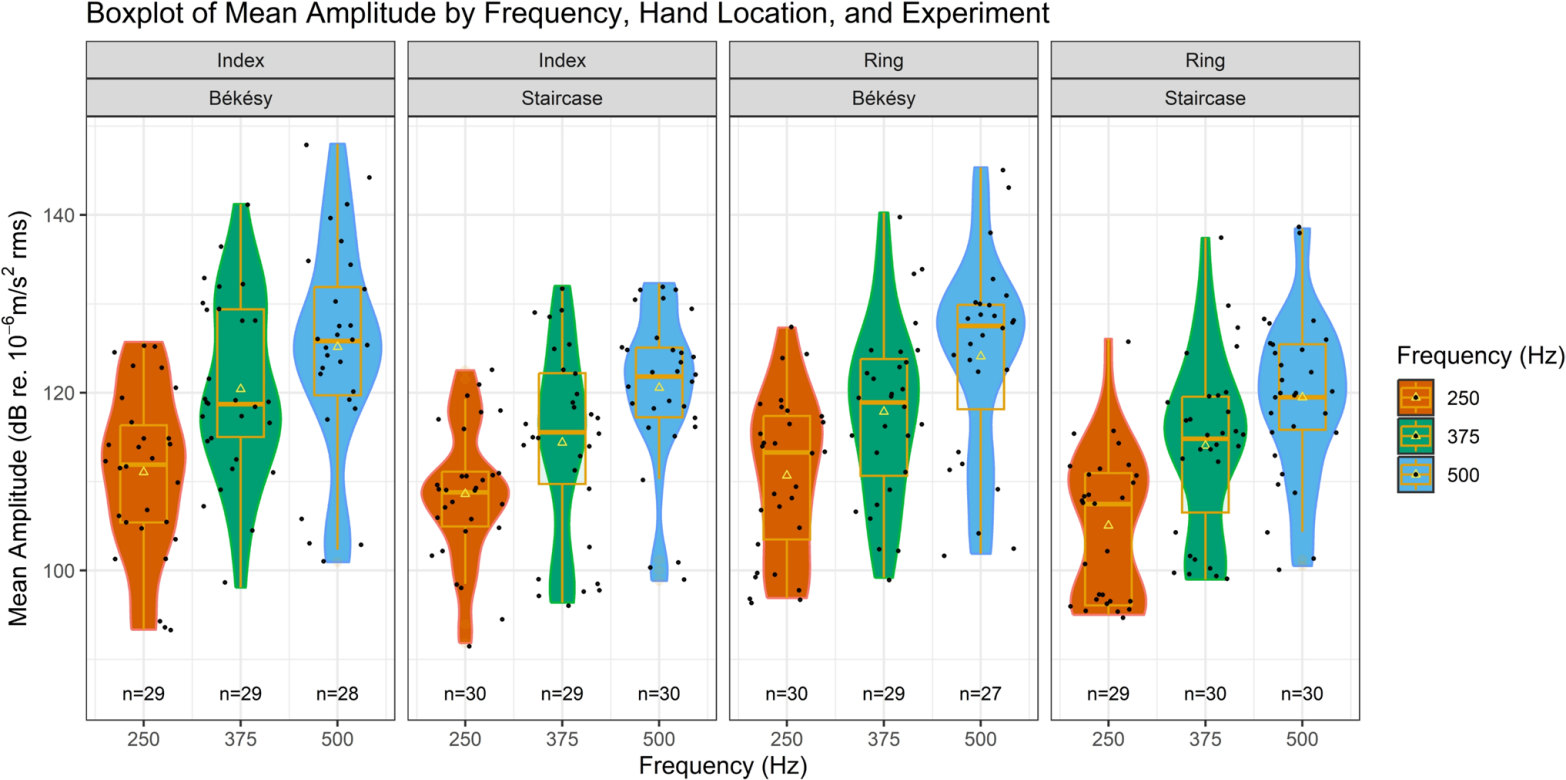
Violin and boxplot of vibration perception thresholds measured at three different frequencies on the finger pulp of the right index and ring fingers of *n* = 30 healthy adults using two separate methods. ∆ represents mean, ●s represent individual participant data, “n” indicates the number of participants that achieved at least eight total reversals for each condition

Table 3 presents a summary of the VPT results obtained from all six conditions, over both methods, where at least eight total reversals occurred. A visual representation is presented in Fig. 6. Information regarding the median and interquartile range of the obtained VPT results is available as Online Supplementary Material. From an analysis of this table and figure, a pattern of increasing VPTs as frequency increases can be noted in the results from both methods, with the Staircase method sporting lower thresholds than the von Békésy method, alongside smaller standard deviation values and tighter minimum and maximum values at each condition.

**Table 3:** Mean vibration perception thresholds (standard deviation) [minimum, maximum] and 95% confidence intervals (CIs) measured at three different frequencies on the finger pulp of the right index and ring fingers of *n* = 30 healthy adults using two separate methods

	Index				Ring			
	von Bekésy	CI	Staircase	CI	von Bekésy	CI	Staircase	CI
250 Hz	110.25 (12.18) [76.48, 128.21]	[108.61, 111.89]	108.61 (8.84) [80.94, 123.33]	[107.31, 109.91]	108.70 (13.13) [78.42, 128.55]	[107.04, 110.35]	105.07 (11.08) [82.32, 128.20]	[103.41, 106.73]
375 Hz	119.45 (13.55) [78.62, 142.33]	[117.56, 121.35]	114.40 (12.99) [83.02, 134.36]	[112.46, 116.34]	117.80 (13.31) [78.00, 141.85]	[116.06, 119.54]	114.04 (12.14) [84.71, 140.64]	[112.26, 115.83]
500 Hz	124.16 (15.52) [82.37, 153.49]	[121.97, 126.35]	120.57 (10.55) [82.41, 134.68]	[119.02, 122.12]	122.56 (15.32) [80.17, 149.72]	[120.45, 124.68]	119.49 (11.36) [84.43, 140.83]	[117.82, 121.16]

*N*Values expressed in dB relative to 10^−6^ m/s^2^

To assess the need to conduct a multilevel analysis, a fit comparison was made between a baseline model, with only intercepts included, and a model allowing intercepts to vary over contexts, i.e., participants (*randomIntercept* model). This comparison showed that the intercepts varied significantly across participants, *X*^2^ (1) = 103.125, *p* <.0001, thus informing the need to conduct multilevel analysis.

Following this, further models were fitted, with the systematic addition of the fixed effects of Frequency, Method, Hand Location, and Gender, as factors (Frequency three levels, all others two levels) to the *randomIntercept* model. The Frequency, Method, and Hand Location fixed effects were added due to being independent variables considered for this study. The Gender fixed effect, in turn, was added to follow Silva, Lisboa, and Costa’s (2024a) recommendation of providing statistical data regarding the presence or absence of significant differences between VPTs obtained from participants of different genders.

Comparing these models revealed that adding the fixed effects of Frequency, *X*^2^ (2) = 180.96, *p* <.001, Method, *X*^2^ (1) = 50.58, *p* <.001, and Hand Location, *X*^2^ (1) = 4.96, *p* =.03, significantly improved the model’s fit, while adding the fixed effect of Gender did not, *X*^2^ (1) = 0.03, *p* =.87. Thus, further examinations will focus on the simplest model that provided the greatest fit to the data, namely the one containing only the fixed effects of Frequency, Method, and Hand Location, with intercepts varying by participants. This model was refitted to the data once again, using the REML method, and is referred to, going forward, as the *Final_VPT_Model*.

Analysis of this model revealed the intercept to correspond to condition Index–250 Hz, von Békésy method. The model’s summary output is presented in Table 4. The effect of vibration frequency was shown to have a moderate (375 Hz compared to 250 Hz) to large (500 Hz compared to 250 Hz) effect on results, with these being, on average, 7.78 dB higher, at 375 Hz, and 13.80 dB higher, at 500 Hz, than at 250 Hz. Significant differences were also found between the two methodologies, with a moderate effect between them. VPT results were an average of 4.91 dB lower when assessed with the Staircase method when compared to results assessed with the von Békésy method. Lastly, the hand location was also shown to be statistically significant, having a small effect on results, with VPTs assessed from the ring finger being an average of 1.47 dB lower than those obtained from the index finger.

**Table 4:** Summary output of the Final_VPT_Model

	b	CI	SE	DF	t-value	p-value	r
**Intercept**	112.17	[109.09, 115.24]	1.54	316	71.78	<.001	0.97
**375 Hz**	7.78	[6.19, 9.36]	0.81	316	9.66	<.001*	0.48
**500 Hz**	13.80	[12.20, 15.39]	0.81	316	17.04	<.001*	0.69
**Staircase method**	−4.91	[−6.22, −3.61]	0.66	316	−7.42	<.001*	0.39
**Ring finger**	−1.47	[−2.77, −0.17]	0.66	316	−2.22	.027**	0.12

* significant at *p* <.01; ** significant at *p* <.05

b and CI values expressed in dB relative to 10^−6^ m/s^2^

b = beta value; CI = 95% confidence interval; SE = standard error; DF = degrees of freedom; r = Pearson correlation coefficient

To confirm if significant differences could also be found between the results from the 375-Hz and 500-Hz frequencies, the *Final_VPT_Model* model was fitted once again, but this time with an intercept corresponding to condition Index–500 Hz (intercept: *b* = 125.96, SE = 1.57, t(316) = 80.28, *p* <.001, *r* = 0.98) instead of Index–250 Hz. Examining its summary output, the effect of frequency of 375 Hz, *b* = −6.02, SE = 0.81, t(316) = −7.42, *p* <.001, *r* = 0.39 was shown to be statistically significant, negative, and having a moderate effect on results, with VPTs at 375 Hz being, on average, 6.02 dB lower than results assessed at 500 Hz.

### VPTs by frequency

Separate multilevel models were fitted specifically for each of the three frequencies, to assess the existence of any further significant differences within each. The intercepts of these three models correspond to the same factors as in the *Final_VPT_Model*, but each for their respective frequency. The summary outputs of these three models are presented in Table 5. Analyzing these summaries, the methodology was shown to have a significant and moderate effect on VPTs across all three models, with results from the Staircase method being lower than those obtained through the von Békésy method. The hand location, in turn, also maintained a consistent trend across all models, with results from the ring finger being lower than those obtained at the index finger. However, this was found to not be statistically significant across all three models, having a small (on the 250-Hz and 375-Hz models) and very small (on the 500-Hz model) effect on results.

**Table 5:** Summary output of the 250-Hz, 375-Hz, and 500-Hz models

	b	CI	SE	DF	t-value	*p*-value	*r*
***250-Hz model***							
**Intercept**	111.72	[108.78, 114.73]	1.5	86	74.67	.0000	0.99
**Staircase method**	−3.87	[−5.76, −1.98]	0.95	86	−4.07	.0001*	0.40
**Ring finger**	−1.78	[−3.67, 0.10]	0.95	86	−1.88	.064	0.20
***375-Hz model***							
**Intercept**	120.28	[116.73, 123.81]	1.78	85	67.57	.0000	0.99
**Staircase method**	−5.31	[−7.77, −2.85]	1.24	85	−4.3	.0000*	0.42
**Ring finger**	−1.54	[−4, 0.91]	1.24	85	−1.25	.2149	0.13
***500-Hz model***							
**Intercept**	125.74	[122.40, 129.43]	1.81	83	69.48	.0000	0.99
**Staircase method**	−5.20	[−7.87, −2.93]	1.27	83	−4.10	.0001*	0.41
**Ring finger**	−1.02	[−3.42, 1.48]	1.26	83	−0.81	.4226	0.09

* significant at *p* <.01

b and CI values expressed in dB relative to 10^−6^ m/s^2^

b = beta value; CI = 95% confidence interval; SE = standard error; DF = degrees of freedom; r = Pearson correlation coefficient

### VPTs by method

Separate multilevel models were fitted specifically for the Staircase and von Békésy methods as well, to assess the existence of any further significant differences between the two methods. The intercepts of both models corresponded to the same factors as in the *Final_VPT_Model* but with their respective methods. The summary outputs of these two models are presented in Table 6. In both models, the effects of 375-Hz and 500-Hz frequencies were both positive and significant in comparison to the intercept of 250 Hz, with 375 Hz having a moderate effect and 500 Hz having a large effect across the two models. The effect of hand location, in turn, was negative on both models, but only significant on the Staircase model. In both models, hand location was shown to have a small effect size.

**Table 6:** Summary output of the Staircase and von Békésy models

	b	CI	SE	DF	*t*-value	*p*-value	*r*
***Staircase model***							
**Intercept**	107.75	[104.59, 110.91]	1.6	145	67.44	.0000	0.98
**375 Hz**	7.13	[5.38, 8.88]	0.89	145	8.05	.0000*	0.56
**500 Hz**	13.02	[11.28, 14.77]	0.88	145	14.77	.0000*	0.78
**Ring finger**	−1.49	[−2.92, −0.07]	0.72	145	−2.07	.0402**	0.17
***von Békésy model***							
**Intercept**	111.69	[108.27, 115.18]	1.8	139	62.13	.0000	0.98
**375 Hz**	8.5	[6.47, 10.75]	1.1	139	7.73	.0000*	0.55
**500 Hz**	14.61	[12.55, 16.91]	1.12	139	13.05	.0000*	0.74
**Ring finger**	−1.36	[−3.07, 0.47]	0.91	139	−1.5	.1371	0.13

* significant at *p* <.01; ** significant at *p* <.05

b and CI values expressed in dB relative to 10^−6^ m/s^2^

b = beta value; CI = 95% confidence interval; SE = standard error; DF = degrees of freedom; r = Pearson correlation coefficient

### VPTs by hand location

Lastly, separate models were also fitted for each of the two hand locations, i.e., right index and ring fingers. The intercepts of both models corresponded to the same factors as in the *Final_VPT_Model*, but with their respective hand locations. The summary outputs of these models are shown in Table 7. From these summaries, the effect of each separate frequency on VPTs was shown to be significantly positive at both locations, increasing as frequency increased. On the Index finger model, both the 375-Hz and 500-Hz frequencies were shown to have a large effect size, while on the Ring finger model, in turn, 500 Hz was shown to also have a large effect size, while 375 Hz only had a moderate effect. The impact of methodology, in turn, was significantly negative, having a moderate effect size on both models.

**Table 7:** Summary output of the Index and Ring finger models

	b	CI	SE	DF	t-value	p-value	R
***Index finger model***							
**Intercept**	112.44	[109.05, 115.83]	1.72	142	65.54	0	0.98
**375 Hz**	7.52	[5.42, 9.61]	1.06	142	7.10	0*	0.51
**500 Hz**	13.34	[11.25, 15.44]	1.06	142	12.60	0*	0.73
**Staircase method**	−4.95	[−6.66, −3.23]	0.87	142	−5.70	0*	0.43
***Ring finger model***							
**Intercept**	110.43	[107.23, 113.64]	1.62	142	68.11	0	0.99
**375 Hz**	7.94	[5.59, 10.3]	1.19	142	6.68	0*	0.49
**500 Hz**	14.15	[11.78, 16.53]	1.20	142	11.77	0*	0.70
**Staircase method**	−4.85	[−6.79, −2.91]	0.98	142	−4.95	0*	0.38

* significant at *p* <.01

b and CI values expressed in dB relative to 10^−6^ m/s^2^

b = beta value; CI = 95% confidence interval; SE = standard error; DF = degrees of freedom; r = Pearson correlation coefficient

### Time to complete (TTC) overview

**Fig. 7:**
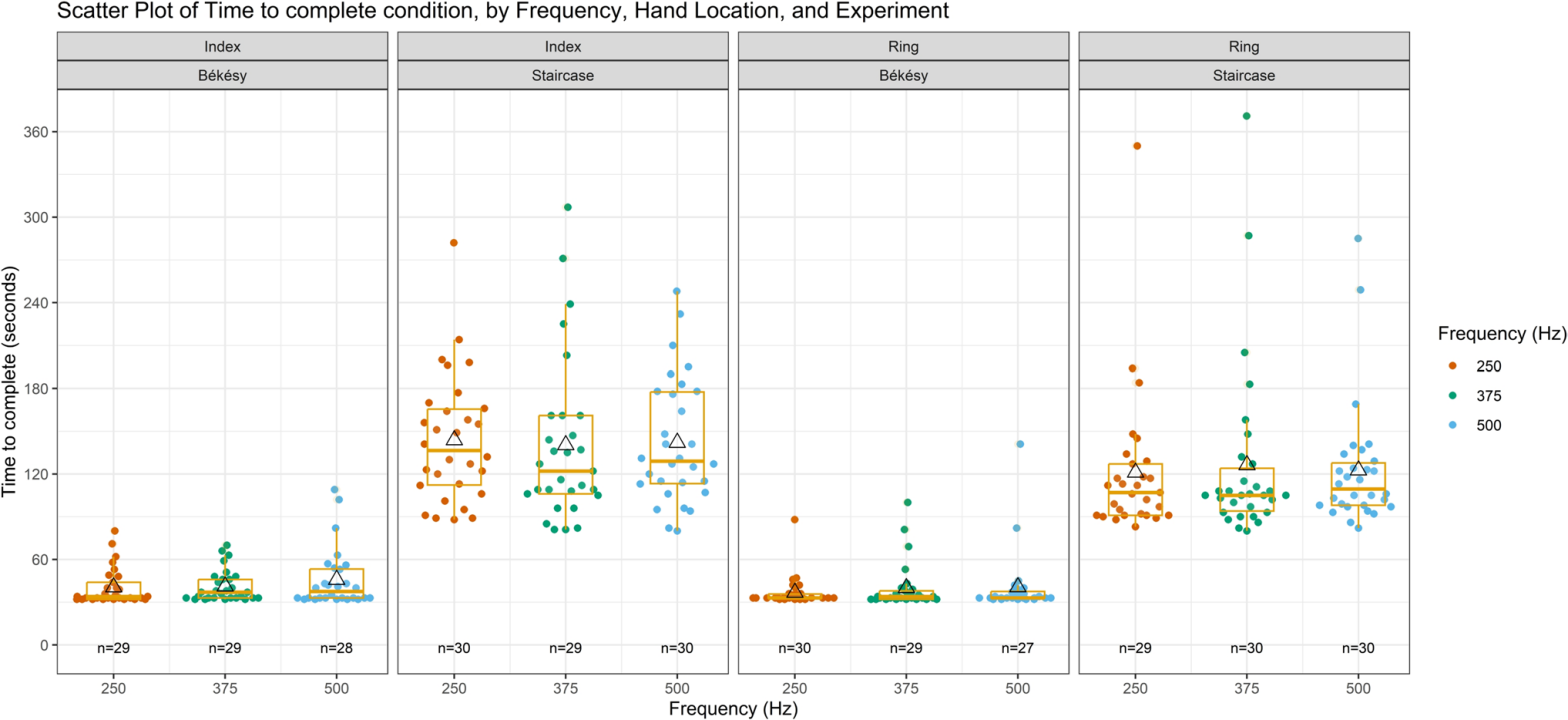
Scatter and boxplot of time to complete conditions involving three different frequencies, two hand locations, and two psychophysical methodologies. Data collected from n = 30 healthy adults. ∆ represents mean, colored ●s represent individual participant data, “n” indicates the number of participants that achieved at least eight total reversals for each condition

Table 8 presents a summary of the TTC results obtained from all six conditions over both methods, where at least eight total reversals occurred. A visual representation is presented in Fig. 7. Information regarding the median and interquartile range of the obtained TTC results is also available as supplementary material. From an analysis of this table and figure, it can be seen that during conditions with the von Békésy method, participants took, in general, less time to achieve eight total reversals when compared to the TTC conditions with the Staircase method. Standard deviation values were also lower with the von Békésy method. It should also be noted that while conditions with the von Békésy method were set to end after eight or more reversals had occurred 30 s from the condition’s onset, due to the HaViThreMa’s software and its update speed, an additional 2 s were added to the ending time. The values presented here were kept with this addition as is for transparency.

**Table 8:** Mean time to complete conditions (standard deviation) [minimum, maximum] in seconds and 95% confidence intervals (CIs) for conditions involving three different frequencies, two hand locations, and two psychophysical methodologies. Data collected from *n* = 30 healthy adults

	Index				Ring			
	von Bekésy	CI	Staircase	CI	von Bekésy	CI	Staircase	CI
250 Hz	41 (13) [32, 80]	[36, 45]	144 (45) [88, 282]	[127, 160]	37 (11) [32, 88]	[33, 41]	121 (52) [83, 350]	[101, 141]
375 Hz	41 (11) [32, 70]	[37, 45]	140 (58) [81, 307]	[118, 162]	40 (16) [32, 100]	[34, 46]	126 (63) [80, 371]	[103, 150]
500 Hz	46 (21) [32, 109]	[38, 54]	142 (44) [80, 248]	[126, 158]	41 (22) [32, 141]	[32, 49]	123 (44) [82, 285]	[106, 139]

Following a similar approach to the model construction for VPT results, to assess the need to conduct a multilevel analysis, a fit comparison was made between a baseline model, with only intercepts included, and a model allowing intercepts to vary over contexts, i.e., participants (*BaseMixModelTime* model). This comparison showed that the intercepts varied significantly across participants, *X*^2^ (1) = 14.01, *p* <.0001, thus informing the need to conduct multilevel analysis.

Given this, further models were fitted, with the systematic addition of the fixed effects of Frequency, Method, Hand Location, and Gender, as factors (Frequency three levels, all others two levels) to the *BaseMixModelTime* model. Model comparison indicated that adding the fixed effects of Method, *X*^2^ (1) = 387.12, *p* <.001, and Hand Location, *X*^2^ (1) = 12.53, *p* <.001, significantly improved the model’s fit, while adding the fixed effect of Frequency, *X*^2^ (2) = 0.51, *p* =.773, and Gender did not, *X*^2^ (1) = 0.261, *p* =.61. Given this, a model was fitted containing only the fixed effects of Method and Hand Location, and a comparison between this model and the *BaseMixModelTime* model revealed it to provide a greater fit to the data, *X*^2^ (3) = 413.412, *p* <.001. Thus, further examinations will focus on this model, which was refitted to the data once again, using the REML method, and is referred to, going forward, as the *Final_Timings_Model*.

Analysis of this model revealed the intercept to correspond to the von Békésy method on the Index finger, *b* = 48.25, SE = 5.21, t(318) = 9.27, *p* <.001, *r* = 0.46. The model’s summary output regarding the fixed effect of Method was *b* = 90.35, SE = 3.26, t(318) = 27.70, *p* <.001, *r* = 0.84, and *b* = −11.60, SE = 3.25, t(318) = −3.56, *p* <.001, *r* = 0.20 for the Hand Location fixed effect. In summary, this result indicates that conditions assessed with the von Békésy method on the index finger took a predicted average of 48.25 s to complete, while conditions with the Staircase method took a predicted average of 90.35 s longer than this to complete, i.e., around 138.6 s on average to complete conditions with the Staircase method. On the other hand, conditions assessed with the von Békésy method on the Ring finger were a predicted average of 11.6 s shorter than those on the Index finger.

## Discussion

The present study was carried out to compare vibration perception threshold (VPT) results assessed at frequencies of 250 Hz, 375 Hz, and 500 Hz, gathered from the pulp of the right index and ring fingers of healthy subjects using the Staircase and von Békésy psychophysical methods. We selected these frequencies for two main reasons. First, Morioka and Griffin (2002) obtained significant differences between VPT results assessed at 125 Hz using the Staircase and von Békésy methods. This raised the question of whether significant differences between results assessed with these two methods could also be found at other frequencies above this value as well. Secondly, our previous systematic review (Silva, Lisboa, & Costa, 2024b) focused on experimental protocols used to assess VPTs at the glabrous skin of the hands and fingers and revealed that far fewer thresholds have been assessed for frequencies between 250 Hz and 500 Hz (seven different frequencies, including) than for those between 4 Hz and 250 Hz (28 different frequencies, not including 250 Hz). Therefore, we also aimed to respond to this gap by contributing information regarding the human perception thresholds at this higher range of frequencies, with the 375 Hz frequency being selected as a middle point between the other two values. Finally, assessments were carried out at the pulps of the right index and ring fingers due to the prevalence of VPT information available in the literature obtained from the former, in contrast to the lack of information obtained from the latter.

### Effect of frequency

With both methods, mean VPT results were found to increase as frequency increased, matching what has been previously found in similar studies that conducted VPT assessments on finger pads with frequencies in the 250- to 500-Hz range (e.g., Ekman et al., 2021; Lundström et al., 2018). Our linear mixed model analysis found significant differences between mean VPT results obtained at these three frequencies on both methods, with mean VPT results at 375 Hz and 500 Hz being, on average, an estimated 7.78 dB and 13.80 dB higher than at 250 Hz, while results at 500 Hz were, on average, an estimated 6.02 dB higher than at 375 Hz.

### Effect of method

The analysis further found significant differences between mean VPT results obtained through both methods, with mean VPT results obtained with the Staircase method across the three frequencies being, on average, 4.91 dB lower than results obtained with the von Békésy method. This effect of lower VPT results being obtained with the Staircase method, following a 3D/1U rule, in comparison to results obtained with the von Békésy method, was also found in Morioka and Griffin (2002), albeit only for 125 Hz, their highest studied frequency. Thus, our study is the first to extend these results to higher vibration frequencies while suggesting that the Staircase method is more accurate than the von Békésy method.

These significant differences between the results from both methods could be for various reasons. For example, ISO 13091-1 (International Organization for Standardization, 2001) informs that exposure to suprathreshold stimuli (i.e., stimuli stronger than a subject’s threshold value) can result in temporary threshold shifts (TTS) during VPT assessments. These shifts can lead to incorrect measurements, as participants will require stimuli to be stronger than normal to be perceptible, thus skewering results upwards. According to ISO 13091-1, the chances of a TTS occurring can be reduced by including an interval between trials, as is usually the case in methods employing intermittent stimulation – hence this standard’s recommendation for employing intermittent stimulation for VPT measurements instead of constant stimulation to avoid TTS. However, including intervals between trials is not feasible in methodologies employing constant stimulation, such as the von Békésy method, so intervals can only be implemented between conditions instead (International Organization for Standardization, 2001). Regarding the effects of TTS on VPT results, Harada and Griffin (1991) showed the effect of TTS on subjects’ VPTs after exposure to different vibrations with an amplitude of 20 ms^−2^ rms (i.e., 146.02 dB re. 10^−6^ m/s^2^) for 5-min periods. Measuring VPTs after exposure, they found that VPT results for frequencies of 250 Hz and 500 Hz deviated the most from control results after exposure to 125 Hz and 250 Hz vibrations, the latter producing the highest deviations.

In our study, the order in which frequencies were presented was randomized, so it can be expected that some subjects were first exposed to vibrations of 250 Hz. Given the results from Harada and Griffin (1991), this may have created a TTS that affected measurements at the other two frequencies. However, vibration exposure during our study, particularly when following the von Békésy method, was neither maintained for long periods at any given amplitude level nor was expected to go greatly beyond each subject’s normal VPTs for each frequency and location. During assessments with this method, vibration amplitude was programmed to either increase or decrease every 1.25 s, thus only being kept at any given amplitude for that long. Furthermore, subjects were instructed to answer “Detected” as soon as they perceived a vibration, with this answer resulting in the vibration’s amplitude being reduced at the next trial. Additionally, each frequency was only tested once at each hand location, and the random distribution of conditions created situations where each location was tested interchangeably between conditions. Lastly, conditions with the von Békésy method took an average of 37–46 s to complete, with the longest running for 141 s (around 2:20 min), less time than the period of exposure used by Harada and Griffin (1991) at a constant frequency and amplitude. Regardless, to further prevent this issue in future studies, authors following a similar approach to our own may choose to restrict conditions assessing thresholds for 250-Hz frequencies to be among the last to be performed. Furthermore, if more than one hand location is being tested, a pattern could also be implemented so that VPTs are not assessed at the same hand location in immediate succession, if conditions allow. Additionally, given the shorter completion time of conditions with the von Békésy method, the inclusion of longer intervals (e.g., 15- to 30-s intervals) between conditions with this method may also be worthwhile to further prevent the occurance of TTS.

The higher VPT results obtained with the von Békésy method could also be related to participants’ reaction times, as suggested by Morioka and Griffin (2002). For reasons such as being momentarily distracted or drowsy, participants might have occasionally taken longer to input their answers, with this delay resulting in their answers at some reversal points being associated with amplitude values higher or lower than intended. To prevent these situations, participants were free to request a break at any point during data gatherings, and although a hard time limit was not imposed on conditions with the von Békésy method, participants did not usually take much longer than 30 s to complete each of them.

Another potential explanation for this difference in threshold results between the two methods could be due to participants, during some trials with the Staircase method, being able to detect the actuator’s ramp-up and/or ramp-down periods when contrasted with the lack of background vibration during intervals. Thus, on trials where they could not perceive a vibration stimulus, they could nevertheless have answered “Detected” after perceiving these occurrences instead, therefore lowering their threshold results. This possibility was also suggested by Morioka and Griffin (2002). To prevent this, the use of actuators with either no ramp-up/ramp-down times or actuators with the capacity to almost immediately reach the full amount of frequency and amplitude inputted would be required, which might not be realistic or cost-effective.

Furthermore, in conditions of both methods, “Detected” and “Not detected” answers may have also been given at times not because subjects did or did not perceive a vibration stimulus, but due to other factors. During conditions with the Staircase method, some answers might have been given because participants realized the rules that the method followed. This recognition could have led them to anticipate the next stimulus, potentially biasing their responses to align with their expectations rather than their actual sensory perception. During conditions with the von Békésy method, in turn, participants may have given answers at some points based on their expectations regarding when they expected to start perceiving or were no longer perceiving the vibration stimuli again, based on the change rate and change direction (increasing or decreasing amplitude) at the time, as well as their expectations regarding their own performance. To prevent these occurrences, researchers should emphasize to participants how important it is that they respond to each trial based on what they are truly perceiving at that point instead of what they think they should be. However, these response biases represent inherent limitations of the two methods.

### Effect of hand location

Our linear mixed model analysis also found significant differences between mean VPT results obtained at the index and ring fingers. From analyzing the summary output of the *Final_VPT_Model*, VPTs assessed at the ring fingers were lower (circa 1.47 dB lower) than the ones measured at the index fingers. This indicates that vibrations might be more easily perceived on the ring finger. However, on further analysis of the models fitted to each of the different factors, this difference was only shown to be significant in assessments carried out with the Staircase method.

Previous research has reported variability in VPTs across different fingers for certain frequencies. However, the pattern regarding which fingers have higher or lower thresholds is not consistent across studies. For example, Ekman et al. (2021), who used the von Békésy method to assess VPTs from seven frequencies on the pulp of the right index and little fingers of subjects, found some significant, albeit small, mean differences between VPTs at the two locations in three of those frequencies. At 16 Hz, mean VPTs on the index finger were higher than on the little finger, while at 32 Hz and 64 Hz, the opposite occurred. Lindsell and Griffin (2003), in turn, compared VPT data assessed from frequencies of 31.5 Hz and 125 Hz on different fingers of either one or both hands. These assessments were conducted at five international test centers, four employing the von Békésy method (Centers 1, 3, 4, and 5) and one using a stepping algorithm (Center 2). Overall, they found results on the middle fingers to be significantly lower than on the little finger of the same hand for both frequencies. However, these significant differences were not consistent across all centers, with results from centers 1 and 4 not showing significant differences between the VPT results for the 31.5-Hz frequency between both fingers. Other studies, in turn, have also reported no significant differences between VPTs obtained at different fingers of the same hand. Dahlin et al. (2015), for example, conducted VPT assessments through the von Békésy method on the pulp of the right index and little fingers for seven different frequencies and found no significant differences in VPTs between the two locations. Similarly, Ahn et al. (2013) also employed the von Békésy method to assess VPTs for frequencies of 31.5 Hz, 125 Hz, and 250 Hz on the index and little fingers of both hands and found no significant differences between the results of either hand or finger.

One potential reason for the threshold differences found in the present study between the index and ring fingers – as well as for the significant differences, or lack thereof, found between the index, middle, and little fingers in the abovementioned studies, among others – where VPT results assessed at the former where significantly higher than at the latter, might be related to the nerves innervating these fingers. Across different individuals, the index finger is solely innervated by the median nerve, while the little finger is only innervated by the ulnar nerve. The ring finger, in turn, can be innervated by both the median and ulnar nerves, or solely by either the ulnar or median nerves, depending on the individual. Similarly, the middle finger can be enervated either by the median nerve only or by the median nerve on its radial side and the ulnar nerve on its ulnar side. In this situation, the ring finger is innervated by the ulnar nerve only (Önne, 1962, as cited in Jones & Lederman, 2006). Thus, some cases may occur where a dual innervation of the ring or middle fingers results in some individuals having an increased sensitivity at these locations in contrast to other people, as overlapping nerve inputs may increase a subject’s sensitivity to stimuli such as vibrations of certain frequencies. This theory, however, requires further and more focused testing, especially since model summary analysis only indicated these differences to be significant for assessments conducted with the staircase method, and were not significant when models were fitted specifically for each frequency and the von Békésy method.

Our findings seem to suggest that the variability observed in previous studies on VPTs may stem from differences in the methodologies used. When comparing two VPT measurement methods on the same participants, we observed discrepancies between the sensitivity of the index and ring fingers only when using the Staircase method. This indicates that the Staircase method may offer a more precise assessment of VPTs, as it can detect subtle sensitivity differences between finger locations, which the von Békésy method did not. These results highlight the importance of method selection in VPT research, as the Staircase method appears to provide finer discrimination in sensitivity across different anatomical locations.

## Limitations and future directions

While the present study provides valuable insights, it is important to acknowledge certain limitations that may have influenced our findings and consider potential avenues for future research to address these challenges and build upon the current results. First, unlike some commercially available instruments for assessiong VPTs at the hands, the Hand Vibration Threshold Mapper (HaViThreMa) (Silva, Lisboa, Matias, et al., 2024) does not possess a means to monitor nor control the participant’s finger skin temperature nor the pressure exerted between the skin and each Piezo Cradle structure, factors that are known to influence VPT assessments (Bolanowski & Verrillo, 1982; Morioka & Griffin, 2005). As the HaViThreMa was developed to be a lightweight and portable instrument to assess VPTs using piezoelectric actuators, the addition of further components, which would have to be added to each cradle, would require not only careful changes to their current design but also proper testing regarding their overall effectiveness and how they interact with the other components already being used. For example, components such as Peltier modules, like those used in Van Riessen and Vardar (2024), could be viable solutions to generate heat at each cradle. However, their inclusion would have to be made in such a way as to not negatively impact the other components already present on each cradle, nor the participant’s skin. Alternatively, the temperature control components could be placed at another location near the skin location where VPTs are to be assessed, as in Van Riessen and Vardar (2024), but the effectiveness of this approach would have to be further studied.

While steps were taken in this study to control skin temperature using commercial hand warmers placed under the palm, variations in skin temperature occurred between the beginning and end of sessions. Furthermore, different skin temperatures were also measured between the two fingers of some participants, both at the start and at the end of a session, with some having a higher temperature on the index finger, and others having a higher temperature on the ring finger instead at either one or both moments. These temperature differences might have impacted the VPT measurements of certain participants who went through larger temperature variations. However, as the primary objective of our study was to compare the two methods (the von Békésy and Staircase methods), and the variations in participants’ skin temperature were minimal between the two methods for each individual, these fluctuations did not significantly impact our main findings.

To measure and control pressure, in turn, components would have to be added that can not only monitor the pressure exerted at each cradle but also report it to the researcher, the participant, or both, in a timely and efficient manner. For example, some studies could benefit from having the exerted pressure at the assessment location be reported alongside each answer to a trial, while keeping participants unaware of how much pressure they are exerting. Other studies, in turn, may need pressure to be kept within a certain range and thus would require participants to know when they are or are not exerting that required amount. While the piezo actuators that the HaViThreMa currently has can monitor pressure, this capability is not available while generating vibrations. A viable solution might be the inclusion of a second layer of actuators at each cradle tasked only with monitoring pressure, but this would require changes not only to the Piezo Cradle’s current design but also to the HaViThreMa’s software, so that researchers, at the very least, can have access to this data.

In the present study, participants were instructed not to exert pressures on each cradle higher than those applied when naturally resting their hands on a surface. Nevertheless, because pressure was not controlled throughout the study, some participants may have occasionally not done so in certain trials/conditions, resulting in some stimuli being perceived more easily or with greater difficulty than they would otherwise. Future studies might benefit from more rigorous control of skin temperature and pressure across testing sessions/trials to better isolate these effects on VPT measurements.

Another limitation is related to the use of the 5% and 2% step sizes/change rates. As our intention was for these to be similar between both methods, an effort was made during pilot testing to assess which values could allow for finer VPT assessments to occur while also accounting for two restrictions. First, step sizes/change ranges could not be so small as to require a large time investment from participants, particularly when the Staircase method was employed. Second, step sizes/change rates could not be so large as to not provide enough time, during assessments with the von Békésy method, for participants with slower reaction times to input their answers at either the intended amplitude level or at a level shortly after, to not skew results. Regardless, the question nevertheless arises regarding how other combinations of initial and follow-up step sizes/change rates could have affected the accuracy of results. For example, it could occur that using smaller step sizes on the Staircase method would result in more accurate threshold values. However, the added time required to complete each condition with smaller step sizes could also generate more drowsiness and thus negatively impact results due to increased distraction. Alternatively, using larger change rates on the von Békésy method could result in either faster assessments, if no minimum time limit was imposed, or in an increased number of total reversals, if no maximum number of reversals was specified. However, results would be greatly dependent on the participant’s reaction times, which could result in less accurate thresholds. Thus, future works may choose to dive into this aspect further, to examine the potential effects that different step sizes and change rates might have on VPT results.

Additionally, the use of an LED to indicate when a condition was running, with the von Békésy method, or when participants could input their answers to a trial, with the Staircase method, may have caused bias in some participants regarding their perception of vibration during a given condition or trial. However, this was a necessary limitation, as we required a method to inform participants of the periods during which they were expected to provide inputs. Without this light, there was a possibility that some participants could be left unsure regarding when it was appropriate to answer, especially during trials with the Staircase method with lower-than-threshold level amplitudes. While an alternative would be to employ auditory cues instead, such cues would run the risk of biasing participants’ answers regardless, and the implementation of such cues, on top of the headphones’ noise-canceling functionality and the masking white noise, alongside the HaViThreMa’s system, would be more cumbersome than the integrated LED light, which is already integrated and controlled by the HaViThreMa software itself. Similar uses of visual stimuli in VPT assessment tasks can be found in studies such as Haseleu et al. (2014), Moshourab et al. (2017), and Prsa et al. (2021). These authors employed a Forced Interval Response type in their assessments, meaning that a vibration was first actuated during one of two or more timed intervals, visually identified on a screen, with participants having to identify at which interval they perceived a vibration. Nevertheless, since our primary objective was to compare the von Békésy and Staircase methods, and the visual cue was applied across both, any observed differences between the two methods cannot be attributed to the presence of the visual signal itself.

Our results showed differences between the VPT results obtained at the three selected frequencies with the employed methods – that is, the Staircase, following a 3 Down/1 Up rule, and the von Békésy methods. This should be taken into account for future studies and implementations, as threshold results assessed through one method may not be comparable to results obtained through other methods. This is particularly relevant when one intends to use threshold information as a basis for decisions such as medical diagnosis or defining actuation parameters for devices with haptic capabilities. For medical diagnosis, VPT data assessed from patients are meant to be compared with available normative data, such as ISO 13091-2 (International Organization for Standardization, 2003), so care must be applied towards ensuring that data assessed from patients was acquired using methods as similar as possible to those used during the assessments that make up the normative data sets, as differences in methodologies can result in significant differences that may hinder diagnostical procedures –regardless, VPT assessments should only be a part of a larger set of diagnostical testing procedures, not the only one through which to base a final diagnosis. Furthermore, based on our and Morioka and Griffin’s (2002) results, when compiling data to create VPT normative data sets, care should be taken not to combine data obtained through these two methods indiscriminately, especially when it comes to data obtained from frequencies above 125 Hz or between 250 Hz and 500 Hz. Averaging results from these two methods may bring normative values down, which could be problematic when data from patients assessed through the von Békésy method are compared to them during diagnostical procedures, which could then be interpreted as being higher than normative, while in actuality not being higher than the normative data obtained through the von Békésy method only, thus hindering these procedures.

Hapticians, in turn, and especially those wishing to conduct their own vibration perception assessments, or wishing to implement said tests in their designs to better match the sensitivity of their users, should also base their selection of which method to implement with the results from this study in mind. For example, while selecting faster assessment methodologies (e.g., the von Békésy method) might be appealing from a time-saving perspective, if these methods provide significantly higher thresholds than more time-consuming methods (e.g., the Staircase method), using said higher thresholds might lead to situations where some participants perceive those higher-amplitude vibrations as uncomfortable. On the other hand, lower thresholds obtained through slower methods might be more accurate, but if new assessments are required due to a change in variables, users may choose to not conduct them due to frustrations with the time required to complete them. Hapticians should not take VPTs as the sole measurement on which to base the decision regarding at which amplitude to set vibration stimuli for users to perceive them. Instead, VPTs should be interpreted as a guideline regarding the minimum value that must be outputted for perception to occur, in contrast with the maximum amount of amplitude that it takes for vibrations to no longer be perceived as comfortable. Thus, VPTs, in haptic feedback technologies, are simply another metric among many others that must not only contend with one another, but are also susceptible to changes due to the different variables that affect one’s ability to perceive vibrations while interacting with devices under various scenarios and conditions (e.g., during exposure to other background vibrations while riding a motorcycle, as in Lisboa et al., 2024, or during exposure to colder temperatures during the winter season).

Lastly, future studies may also opt to conduct further comparisons between VPT results obtained through different psychophysical methodologies – such as the method of limits (Kingdom & Prins, 2016) or the psi-marginal adaptative algorithm (Prins, 2013), which have also been used in prior literature to assess these thresholds (Gandhi et al., 2011; Silva, Lisboa, & Costa, 2024b) – to assess if significant differences between results from different methods might not also be found at other frequencies.

## Conclusion

Vibration perception threshold (VPT) assessments were carried out at frequencies of 250 Hz, 375 Hz, and 500 Hz, using the Staircase and von Békésy methods, on the pulp of the right index and ring fingers. As frequency increased, VPTs also increased. This relation was found in both methods and in both hand locations.

The Staircase method following the 3 Down/1 Up targets the 79.4% point of the psychometric curve, while the von Békésy method targets the 50% probability instead. While the expectation would be for VPTs assessed with the former to be higher than those assessed with the latter, the opposite was found across all three frequencies, with an average difference of approximately 4.9 dB between the two methods, which was found to be statistically significant. This suggests that the Staircase method may be more sensitive in detecting lower vibration perception thresholds despite the higher probability target, which could mean that it is more effective in identifying subtle differences in tactile perception, potentially offering a more accurate measurement of how vibrations are perceived at varying intensities at the cost of longer assessment runtimes. Further testing is nevertheless required to explore and better understand these differences, as the use of different Up and Down rules and step sizes, as well as different total condition run times and change rates, may influence these results at the assessed frequencies. Additionally, studies comparing whether these differences remain consistent upon repeated measurements across separate weeks or months would also be beneficial to assess if the patterns found in this study remain consistent over those periods, which would also provide more information regarding these methods’ effectiveness and accuracy.

Significant differences between VPT results across genders were not found. Results obtained at the ring finger were also lower than those obtained at the index finger, though this effect was only significant for results obtained with the Staircase method. Conditions also took less time to complete when conducted at the ring finger (estimated average of 36.65 s with the von Békésy method) than when conducted at the index finger (estimated average of 48.25 s with the von Békésy method). This may be due to a potential higher sensitivity at the pad of this finger, resulting in participants requiring fewer total trials to achieve at least eight total reversals, and thus lower TTC for conditions on this location.

Additionally, and as expected, data assessments conducted through the von Békésy method took less time to complete (estimated average of 48.25 s on the index finger) than those conducted through the Staircase method (estimated average of 138.6 s on the index finger). Thus, given this and the Staircase method’s higher sensitivity and accuracy when assessing VPTs, it might be more advantageous to conduct assessments with this method when the number of total assessments is relatively low, as the added time might be compensated through better and more reliable data. On the other hand, if we take the estimated average TTC, in the time it would take to complete one assessment with the Staircase method, around three assessments could be completed with the von Békésy method instead. However, strategies exist that can help shorten the TTC of assessments with the Staircase method, such as reducing the number of required reversals, decreasing the number of consecutive “detected” responses to lower the stimulus amplitude, and/or removing the “consecutive answers” requirement all together, though these would also warrant comparisions to assess their effects on the obtained data in comparison to the method employed here.

Thus, we conclude that the choice of which psychophysical methodology to employ should be carefully considered when intending to assess VPTs at the pulp of the fingers. This is especially true when the assessment results are meant to be used directly to lead decisions, such as haptic design choices, or to be compared to previously gathered normative data from other sources and/or methodologies for diagnostical purposes.

## Supplementary Information

Below is the link to the electronic supplementary material.

Supplementary file1 (XLSX 19 KB)

Supplementary file2 (XLSX 15 KB)

## Data Availability

All data related to the study presented in this paper are available at the Open Science Framework (OSF), at the following URL: https://osf.io/3uqsb/overview?view_only=03e228c15a274cb781045e3b61ccb052.

## Code availability

The analysis code used to conduct the analysis presented in this paper is available at the OSF**, **at the following URL: https://osf.io/3uqsb/overview?view_only=03e228c15a274cb781045e3b61ccb052.

## Declarations

### Open practices statement

The data and code files related to the study presented in this paper are available at the Open Science Framework (OSF), at the following URL: https://osf.io/3uqsb/?view_only=4e​3235e88a134dbc89aa8df4e3c385f0. This study was not preregistered.
